# Supplementary material for: Association between Eruption Sequence of Posterior Teeth, Dental Crowding, Arch Dimensions, Incisor Inclination, and Skeletal Growth Pattern
Source: Children (Basel). 2023 Apr 1;10(4):674. doi: 10.3390/children10040674 (PMC10137228; doi:10.3390/children10040674)
Supplement: Supplementary file 1 [file children-10-00674-s001.zip › Supplementary Table S1.pdf]

		Total arch space (mm)	Space 5_4_3 (right) (mm)	Space 2_1_1_2 (mm)	Space 3_4_5 (left) (mm)	TS-ALD (mm)	Tooth size 5_4_3 (right) (mm)	Tooth size 2_1_1_2 (mm)	Tooth size 3_4_5 (left) (mm)	Anterior arch length (mm)	Posterior arch length (mm)	Total arch length (mm)	Intermolar width (mm)	Intercanine width (mm)	Inclination Upper Incisor_NA (º)	Distance Upper Incisor_NA (mm)	ANB angle (º)	Wits (mm)	Mandibular plane (º)	Lower Facial Height (º)
Total arch space (mm)	r	1	.655**	.436**	.707**	.373**	.449**	.290**	.468**	.373**	.419**	.588**	.598**	.634**	.138	.242*	.114	.064	-146	.111
	Sig. (bilateral)		.000	.000	.000	.001	.000	.001	.000	.001	.000	.000	.000	.000	.218	.030	.309	.570	.195	.330
Space 5_4_3 (right) (mm)	r	.655**	1	-.041	.591**	.556**	.243*	-.036	.265*	.266*	.298**	.419**	.504	.529**	.058	.114	.036	-.074	-.176	-.013
	Sig. (bilateral)	.000		.714	.000	.000	.029	.749	.170	.016	.007	.000	.000	.000	.609	.311	.747	.513	.116	.911
Space 2_1_1_2 (mm)	r	.436**	-.041	1	.016	-.077	.325**	.617**	.354**	.152	.343**	.368**	.214	.267	-.014	.141	.188	.109	.173	.082
	Sig. (bilateral)	0,000	0,714		0,888	.116	.003	.000	.001	.176	.002	.001	.055	.061	.902	.208	.093	.332	.123	.467
Space 3_4_% (left)	r	.707**	.591**	.016	1	-.177	.325**	.617**	.354**	0,152	.343**	.368**	.214	.267	-.014	.141	.188	.109	.173	.082
	Sig. (bilateral)	.000	.000	0,888		.000	.000	.849	.001	.000	.091	.000	.000	.001	.525	.477	.632	.773	.392	.065
TS-ALD (mm)	r	,373**	,566**	-.177	.470**	1	-.450*	-.281*	-.362**	.315**	.004	.241*	.325**	.047	.049	-.032	.089	.048	-.167	-.002
	Sig. (bilateral)	0,001	0,000	116	.000		.000	.012	.001	.004	.969	.031	.003	.749	.668	.778	433	.670	.140	.998
Tooth size 5_4_- (right) (mm)	r	.449**	.243*	.325**	.393**	-.450**	1	.222*	.894**	.061	.381**	.326**	.251*	.452**	.042	.153	-.052	-.157	.041	-.157
	Sig. (bilateral)	.000	.019	.003	.000	.000		.046	.000	.589	.000	.003	.024	.001	.710	.172	.646	.163	.718	.163
Tooth size 2_1_1_2 (mm)	r	.290**	-.036	.617**	.022	-.281*	.222*	1	.326**	.128	.244*	.277*	.115	.233	.055	.164	.099	.123	.048	.054
	Sig. (bilateral)	.009	.749	.000	.849	.012	.046		.003	.254	.028	.120	.307	.104	.629	.144	.379	.274	.670	.629
Tooth size 3_4_5 (left) (mm)	r	.468**	.265*	.354**	.364**	-.362**	.894**	.326**	1	.104	.361**	.344**	.252*	.503**	.089	.142	-.024	-.093	-.006	-.212
	Sig. (bilateral)	.000	.017	.001	.001	.001	.000	.003		.357	.001	.002	.023	.000	.430	.205	.831	.408	.959	.058
Anterior arch length (mm)	r	.373**	.266*	.152	.448**	.315**	.061	.128	.104	1	-.103	.699**	.149	.134	.375**	.344**	.055	.047	.081	-.009
	Sig. (bilateral)	.001	.016	.176	.000	.004	.589	.354	.357		.360	.000	.184	.352	.001	.002	.250	.678	.472	.938
Posterior arch length (mm)	r	.419**	.298**	.343**	.189	.004	.381**	.244*	.361**	-.103	1	.671**	.345	.333*	-.148	-.048	-.028	.029	-.331*	-.053
	Sig. (bilateral)	.000	.007	.002	.091	.969	.000	.028	.001	.360		.000	.002	.018	.189	.674	.806	.800	.003	.637
Total arch length (mm)	r	.588*	.419**	.368**	.474	.241*	.326**	.277	.344**	.660**	.671**	1	.369**	.346*	.167	.218	.016	.055	-.184	-.047
	Sig. (bilateral)	.000	.000	.001	.000	.031	.003	.012	.002	.000	.000		.001	.014	.137	.050	.886	.624	.101	.679
Intermolar width (mm)	r	.598**	.504**	.214	.459**	.325**	.251*	.115	.252*	.149	.345**	.369**	1	.572**	0,029	.101	-.196	-.206	-.061	-.063
	Sig. (bilateral)	.000	.000	.005	.000	.003	.024	.307	.023	.184	.002	.001		.000	.794	.367	.077	.063	.586	.575
Intercanine width (mm)	r	.634**	.529**	.267	.464**	.047	.052**	.233	.503**	.134	.333*	.346*	.572**	1	.097	.238	-.221	-.114	-.355*	-.296*
	Sig. (bilateral)	.000	.000	.061	.001	.749	.001	.104	.000	.352	.018	.014	.000		.502	.096	.122	.429	.011	.037
Inclination Upper Incisor_NA (º)	r	.138	.058	-.014	-.072	.049	.042	.055	.089	.375**	-.148	.167	.029	.097	1	.742**	-.306	-.078	-.058	-.158
	Sig. (bilateral)	.218	.609	.902	.525	.668	.710	.629	.430	.001	.189	.137	.794	.502		.000	.002	.439	.569	.117
Distance Upper Incisor_NA (mm)	r	.242*	.114	.141	.086	-.032	.153	.164	.142	.344**	-.048	.218	.101	.238	.742**	1	-.247*	-.133	.097	.007
	Sig. (bilateral)	.030	.311	.208	.447	.778	.172	.144	.205	.002	.674	.050	.367	.096	.000		.013	.187	.336	.942
ANB (º)	r	.114	.036	.188	.054	.089	-52	.099	-.024	.055	-.028	.016	-.196	-.221	-.306**	-.247*	1	.576**	.221*	.320**
	Sig. (bilateral)	.309	.747	.093	.632	.433	.646	.379	.831	.625	.806	.886	.077	.122	.002	.013		.000	.027	.001
Wits (mm)	r	.064	-.074	.109	-.032	.048	-.157	.123	-.093	.047	.029	.055	-.206	-.144	-.078	-.133	.576**	1	-.123	-.032
	Sig. (bilateral)	.570	.513	.332	.773	.670	.163	.274	.408	.678	.800	.624	.063	.429	.439	.187	0,000		.222	.754
Mandibular plane (º)	r	-.146	-.176	.173	-.096	-.167	.041	.048	-.006	.081	-.331**	-.184	-.061	-.355	-.058	.097	.000	-.123	1	.432**
	Sig. (bilateral)	.195	.116	.123	.392	.140	.718	.670	.959	.472	.003	.101	.586	.011	.569	.336	.027	.222		.000
Lower Facial Height (º)	r	-.110	-13	.082	-.206	-.002	-.157	.054	-.212	-.009	-.053	-.047	-.063	-.296*	-.158	.007	.320**	-.032	.432**	1
	Sig. (bilateral)	.330	.911	.467	.065	.988	.163	.629	.058	.938	.637	.679	.575	.037	.117	.942	.001	.754	.000	

Figure S1. Pearson Correlation coefficients (r) for outcomes measured in the UPPER ARCH. Sig: p value, two-sided.
