# Supplementary material for: Association between Eruption Sequence of Posterior Teeth, Dental Crowding, Arch Dimensions, Incisor Inclination, and Skeletal Growth Pattern
Source: Children (Basel). 2023 Apr 1;10(4):674. doi: 10.3390/children10040674 (PMC10137228; doi:10.3390/children10040674)
Supplement: Supplementary file 1 [file children-10-00674-s001.zip › Supplementary Table S2.pdf]

		Total arch space (mm)	Space 5_4_3 (right) (mm)	Space 2_1_1_2 (mm)	Space 3_4_5 (left) (mm)	TS-ALD (mm)	Tooth size 5_4_3 (right) (mm)	Tooth size 2_1_1_2 (mm)	Tooth size 3_4_5 (left) (mm)	Anterior arch length (mm)	Posterior arch length (mm)	Total arch length (mm)	Intermolar width (mm)	Intercanine width (mm)	Inclination Upper Incisor_NA (º)	Distance Upper Incisor_NA (mm)	ANB angle (º)	Wits (mm)	Mandibular plane (º)	Lower Facial Height (º)
Total arch space (mm)	r	1	.653**	.298*	.760**	.158	.457**	.368*	.450**	.271	.212	.402**	.284	.277	.159	.235	.135	.080	-.095	-.039
	Sig. (bilateral)		.000	.047	.000	.283	.001	.010	.001	.062	.147	.005	.053	.108	.280	.108	.360	.591	.019	.790
Space 5_4_3 (right) (mm)	r	,653**	1	.167	.809**	.419**	.327	.249	.317*	.327*	.302*	.522**	.258	.176	.187	.065	-.048	-.960	-.209	.041
	Sig. (bilateral)	.000		.256	.000	.003	230	.088	.028	.023	.037	.000	.080	.312	.203	.660	.747	.517	.153	.783
Space 2_1_1_2 (mm)	r	.298*	.167	1	.137	-.016	.374**	.559**	.391*	.100	.266	.272	.186	.610**	.227	.097	.029	-.066	.131	-.050
	Sig. (bilateral)	.047	.256		.351	.913	.009	.000	.006	500	.122	.062	.211	.000	.121	.512	.847	.657	.376	.735
Space 3_4_% (left)	r	.760**	.809**	.138	1	.380**	.344*	.367*	.334	.262	.304*	.471**	.255	.308	.193	.148	.103	.035	-.123	-.014
	Sig. (bilateral)	.000	.000	.351		.008	.017	0,010	.020	.072	.036	.001	.084	.072	.189	.314	.492	.814	.405	.025
TS-ALD (mm)	r	.158	.419**	-.016	.380**	1	-.554**	-.293*	-.555*	.244	-.137	.085	.083	.098	-.022	.140	-.128	.075	-.287*	.230
	Sig. (bilateral)	.283	.003	.913	.008		.000	.043	.000	.095	.353	.565	.578	.574	.880	.342	.384	.612	.048	.116
Tooth size 5_4_· (right) (mm)	r	.457**	.327*	.374**	.344*	-.554**	1	.472**	.987*	-.049	.391**	.289*	.118	.086	.139	.215	.128	-.004	.052	-.296*
	Sig. (bilateral)	.001	.023	.009	.017	.000		.001	.000	.739	.006	.046	.431	.622	.346	.143	.384	.977	.728	.041
Tooth size 2_1_1_2 (mm)	r	.368*	.249	.559**	.367*	-.293*	.472**	1	.469**	.015	.487**	.420**	.293*	.487**	.332	.252	.228	-.016	.148	.057
	Sig. (bilateral)	.010	.088	.000	.010	.043	.001		.001	.918	.000	.003	.046	.003	.021	.084	.119	.914	.315	.702
Tooth size 3_4_5 (left) (mm)	r	.450**	.317*	.391**	.334	-.555**	.987	.469**	1	-.076	.395**	.270	.108	.110	.131	.195	.119	-.033	.080	-.261
	Sig. (bilateral)	.001	.028	.006	.020	.000	.000	.001		.609	.006	.063	.047	.531	.376	.185	.422	.824	.588	.073
Anterior arch length (mm)	r	.271	.327*	.100	.262	.244	-.049	.015	-.076	1	-.279	.589**	.077	.126	.412**	.290*	.011	-.094	-.175	-.131
	Sig. (bilateral)	.062	.032	.500	.071	.095	.739	.918	.609		.055	.000	.605	.472	.004	.046	.943	.527	.235	.373
Posterior arch length (mm)	r	.212	.302*	.226	.304*	-.137	.391**	.487**	.395**	-.279	1	.612**	.273	.330	.084	.266	.156	.067	.092	.222
	Sig. (bilateral)	.147	.037	.122	.036	.353	.006	.000	.006	.055		.000	.063	.053	.570	.068	.289	.650	.533	.129
Total arch length (mm)	r	.402**	.522**	.272	.471**	.085	.289*	.420**	.270	.589**	.612**	1	.290*	.328	.409**	.463**	.138	-.021	-.066	.078
	Sig. (bilateral)	.005	.000	.062	.001	.565	.046	.003	.063	.000	.000		.048	.054	.004	.001	.348	.885	.655	.597
Intermolar width (mm)	r	.284	.258	.186	.255	.083	.118	.293*	.108	.077	.273	.290*	1	.201	.078	.159	.119	-.029	.255	.292*
	Sig. (bilateral)	.045	.080	.211	.084	.578	.431	.046	.470	.605	.063	.048		.247	.601	.285	.427	.846	.084	.046
Intercanine width (mm)	r	.277	.176	.610**	.308	.098	.086	.487**	.110	.126	.330	.328	.201	1	.155	-.008	.192	.047	-.144	.081
	Sig. (bilateral)	.108	.312	.000	.072	.574	.622	.003	.531	.472	.053	.054	.247		.375	.965	.269	.786	.410	.642
Inclination Upper Incisor_NA (º)	r	.159	.187	.227	.193	-.022	.139	.332*	.131	.412**	.840	.409**	.078	.155	1	.299**	.341**	.118	-.028	-.001
	Sig. (bilateral)	.280	.203	.121	.1i89	.880	.346	.021	.376	.004	.570	.004	.601	.375		.002	.001	.242	.783	.992
Distance Upper Incisor:NA (mm)	r	.235	.065	.097	.148	-.140	.215	.252	.195	.290*	.266	.463**	.159	-.008	.299**	1	.306**	.124	.105	.180
	Sig. (bilateral)	.108	.660	.512	.314	.342	.143	.084	.185	.046	.068	.001	.285	.965	.002		.002	.218	.298	.073
ANB (º)	r	.135	-.048	.029	.102	-.128	.128	.228	.119	.011	.156	.138	.119	.192	.341**	.306**	1	.576**	.221*	.320**
	Sig. (bilateral)	.360	.747	.847	.492	.384	.384	.119	.422	.943	.289	.348	.427	.269	.001	.002		.000	.027	.001
Wits (mm)	r	.080	-.096	-.066	.035	.075	-.004	-.016	-.003	-.094	.067	-.021	-.029	.047	.118	.124	.576**	1	-0,123	-.032
	Sig. (bilateral)	.591	.517	.657	.814	.612	.977	.914	.824	.527	.650	.885	.846	.786	.242	.218	.000		.222	.654
Mandibular plane (º)	r	-.095	-.209	.131	-.123	-.287*	.052	.148	.080	-.175	.092	-.066	.255	-.144	-.028	.105	.221*	-.123	1	.432**
	Sig. (bilateral)	.519	.153	.376	.405	.048	.728	.315	.588	.235	.533	.655	.084	.410	.783	.298	.027	.222		.000
Lower Facial Height (º)	r	-.039	.041	-.050	-.014	.230	-.296*	.057	-.261	-.131	.222	.078	.292*	.081	-.001	.180	.320**	-.320	.432**	1
	Sig. (bilateral)	.790	.783	.735	.925	.116	.041	.702	.073	.373	.129	.597	.046	.642	.992	.073	.001	.754	.000	

Figure S2. Pearson Correlation coefficients (r) for outcomes measured in the LOWER ARCH. Sig: p value, two-sided.
